# Superfund: Evaluating the Impact of Executive Order 12898

**DOI:** 10.1289/ehp.9903

**Published:** 2007-04-05

**Authors:** Sandra George O’Neil

**Affiliations:** Curry College, Milton, Massachusetts, USA

**Keywords:** environmental justice, Executive Order 12898, National Priorities List, Superfund

## Abstract

**Background:**

The U.S. Environmental Protection Agency (EPA) addresses uncontrolled and abandoned hazardous waste sites throughout the country. Sites that are perceived to be a significant threat to both surrounding populations and the environment can be placed on the U.S. EPA Superfund list and qualify for federal cleanup funds. The equitability of the Superfund program has been questioned; the representation of minority and low-income populations in this cleanup program is lower than would be expected. Thus, minorities and low-income populations may not be benefiting proportionately from this environmental cleanup program. In 1994 President Clinton signed Executive Order 12898 requiring that the U.S. EPA and other federal agencies implement environmental justice policies. These policies were to specifically address the disproportionate environmental effects of federal programs and policies on minority and low-income populations.

**Objective and Methods:**

I use event history analysis to evaluate the impact of Executive Order 12898 on the equitability of the Superfund program.

**Discussion:**

Findings suggest that despite environmental justice legislation, Superfund site listings in minority and poor areas are even less likely for sites discovered since the 1994 Executive Order.

**Conclusion:**

The results of this study indicate that Executive Order 12898 for environmental justice has not increased the equitability of the Superfund program.

In the early 1980s environmental justice became widely recognized, as minority and low-income neighborhoods fought to keep environmental hazards out of their communities and away from their families. Researchers began to analyze and document environmental injustices around the country and found that minorities and poor were in fact, more frequently living near environmental hazards [Bullard 1983; United Church of Christ (UCC) 1987; U.S. Government Accounting Office (U.S. GAO) 1983]. As the environmental justice movement began to grow in strength, the U.S. Congress and President Carter established the Comprehensive Environmental Response Compensation and Liability Act or CERCLA (1980). As part of CERCLA and in an effort to address hazardous waste sites across the country, the U.S. Environmental Protection Agency (EPA) must locate and prioritize the most severe sites for remedial action. Sites perceived to be the most threatening to both surrounding populations and the environment can be placed on the National Priorities List (also known as NPL or Superfund list; U.S. EPA 2007) and are then eligible to receive funding through the Superfund. Although the specific ways in which toxins in the ground, water, or air produce adverse health effects in humans are still disputed, the toxicity of such sites and potential risks posed to human populations and the environment certainly warrant attention. As of March 2003, there were 1,484 sites on the Superfund list, and over 6.5 million people living in census tracts with Superfund sites (O’Neil 2005).

Ideally the process of moving a site to the federal Superfund list would be related solely to the severity of hazard posed to the surrounding populations. However, other social forces shape the listing of a Superfund site. A site could be listed because of its hazardousness, or conversely it could be listed because it is less hazardous and therefore easier to clean (Daley and Layton 2004). Moreover, a site could also move more quickly through the listing process because it is in a community perceived to have more power through access to resources because individuals residing in the area have higher incomes or because of racial or ethnic composition of the area. This environmental cleanup injustice has been supported by research demonstrating that representation of minorities and low-income populations is lower in areas with Superfund sites, indicating these populations are not benefiting equally from the Superfund program (Anderton et al. 1997; Hird 1993; O’Neil 2005). In this article I describe the analysis of how of a hazardous site is placed on the Superfund list and further evaluate the effects of legislation intended to make such processes more equitable.

In February 1994, in an attempt to remedy environmental injustice, President Clinton established Executive Order 12898. The order required that

each Federal agency shall make achieving environmental justice part of its mission by identifying and addressing, as appropriate, disproportionately high and adverse human health or environmental effects of its programs, policies, and activities on minority populations, and low-income populations. . . . (Clinton 1994)

This order clearly aims to rectify environmental problems that have disproportionately affected minority and low-income populations. The order specifically names these populations and requires that agencies address policies and programs that may adversely affect these populations in particular. However, the order and its implementation have been criticized [Murphy-Green and Leip 2002; U.S. Government Accountability Office 2005 (the U. S. Government Accounting Office changed its name in July 2005 to the U.S. Government Accountability Office, but the acronymn remained the same as before); U.S. Office of the Inspector General (U.S. OIG) 2004]. The degree to which the order has been both defined and administered at the federal level has been questioned.

In this present study, I evaluate the effects of Executive Order 12898. It has now been over a decade since the executive order, yet the effect of this order on programs such as Superfund has not been critically examined. This historical, national, census tract–level study adds important new information to the field of environmental justice by examining justice in environmental remediation, also known as environmental cleanup justice. The main questions in the present study are *a*) what demographic factors are associated with the chances of being placed on the Superfund list, and *b*) has the executive order affected the chances of listing for minority and poor populations? This study includes U.S. EPA Hazardous Ranking Scores (HRS) and other site-specific data, four decades of demographic information, gender and family variables, and event history analysis with time-varying covariates. This is the first evaluation of the equitability of Superfund listings since Executive Order 12898 and will add significantly to environmental cleanup justice theory as well as policy debate regarding both Superfund and the executive order.

## Environmental Cleanup Justice

Environmental justice argues that the burden of hazardous waste has been unevenly distributed, with heavier burdens on those with less power, of lower socioeconomic status, and people of color. This present article includes studies done by the U.S. GAO (U.S. GAO 1983), the United Church of Christ (UCC 1987), and Robert Bullard’s study of Houston, Texas (Bullard 1983), and concludes that minorities are overburdened by environmental hazards. Although some researchers have asserted that the disproportionate burden of waste is solely associated with income (Bowen et al. 1995; Szasz 1994), other researchers have supported (to varying degrees) the claim that both minorities and the poor are disproportionately affected by environmental burdens (Anderton et al. 1994a, 1994b; Been 1993, 1994; Been and Gupta 1997; Bullard 1993; Bullard et al. 1994; Daniels and Friedman 1999; Downey 1998; Faber and Krieg 2001; Krieg 1995; Logan and Molotch 1987; Mohai and Bryant 1992).

Athough there is an abundance of research on environmental burdens and environmental justice, far fewer studies examine the relationship between environmental remediation and environmental justice, or environmental cleanup justice. The distinction between these two concepts has not been clearly demarcated. Environmental justice and cleanup justice studies have been combined indiscriminately, using Superfund as an indicator of environmental equity (Downey 2005; Krieg 1998; Stretesky and Hogan 1998). However, this assumes that Superfund sites are similar to other environmental hazards such as toxic releases, incinerators, and other hazardous facility sitings. Superfund is and should be a distinctly different analysis in environmental justice. Unlike general environmental justice studies regarding disproportionate exposure or proximity to hazards, the Superfund program is an environmental cleanup program that requires resources and symbolizes an environmental benefit. The cleanup of hazardous waste sites is enormously costly, and therefore clean environments take up limited resources. These resources, not unlike other limited resources, may not be distributed evenly throughout the population, and it is likely that minorities and low-income groups are under-represented in such programs. Furthermore, hazardous sites are not automatically placed on the NPL. Sites may be discovered and never proposed to the Superfund list, or sites may be proposed to the Superfund list but never make the list. Therefore, environmental cleanup justice would require that minorities and the poor be treated the same as other socioeconomic groups, represented proportionately, and encouraged to participate in the cleanup of environmental hazards in their communities to the same or even greater extent than other populations.

Although limited, previous research on nationwide Superfund equity has focused largely on representational equity, the degree to which populations are proportionately represented in the Superfund program (Anderton et al. 1997; Hird 1993; Zimmerman 1993). This small group of Superfund equity research includes a study claiming that minority and poor populations are found in higher percentages in communities with NPL sites (Zimmerman 1993). This suggests that Superfund mimics traditional environmental justice literature, which shows that minorities are overrepresented in proximity to environmental burdens and are also overrepresented in the Superfund program. However, Zimmerman’s research excluded rural sites, and because of the strong association between urban environments and minority and poor populations, the exclusion of these sites may have severely biased results (Anderton et al. 1997; O’Neil 2005). Further research using a national sample and county-level data found inconsistent results and reported that although the poor were less likely to be represented in Superfund and the wealthy more likely to be represented in Superfund—as environmental cleanup justice would suggest—minorities were in fact also more likely to live near such sites (Hird 1993). Therefore, testing of the environmental justice hypothesis has, thus far, produced mixed results. A national census-tract level, Superfund equity study (Anderton et al. 1997) further supported the idea that the wealthiest as well as the poorest were excluded from Superfund and concluded that census tracts with Superfund sites “are typically working class communities with fewer minorities, are less densely populated, and have more industrial employment.” This study also concludes that as

the percentage of Blacks or Hispanics and socioeconomically disadvantaged families increases in a neighborhood, the risk that CERCLIS [Comprehensive Environmental Response and Liabilities Information System database] sites will be placed on the NPL decreases. (Anderton et al. 1997)

This clearly supports the environmental cleanup justice theory. Additionally, a ZIP code-level study claimed that Superfund sites in minority and poor areas take longer to reach the NPL and subsequently take longer to be cleaned. These authors also concluded that cleanups at sites in these areas are also not enforced to the same degree as in white or wealthier areas (Lavelle and Coyle 1992). Therefore, studies up to this point have shown mixed results regarding which populations are most likely to benefit from the Superfund program. However, there is evidence that the poor, and in some cases minorities, are underrepresented in the Superfund program and may not be benefiting equally from environmental cleanups.

## Superfund at Zero

The Superfund program has been under intense scrutiny and criticism since its inception. When the program was created, there is no doubt that the depth, breadth, and complexity of the hazardous waste problem in our nation was seriously underestimated. It did not take long for critics to begin citing how the Superfund program had failed the nation. It was believed that the cleanups were taking too long, there were too few of them, and they were taking too many resources. Tracking progress for so many complicated projects was certainly a challenge (Probst and Sherman 2004). The program could be further criticized for not adequately addressing the potential risks of sites and not prioritizing site cleanups based on potential risk [Environmental Law Institute (ELI) 1999]. In the first 10 years of the program, the U.S. EPA discovered over 1,400 potential Superfund sites (sites that were discovered and met hazard requirements for listing) but deleted only 25 (O’Neil 2005). However, the U.S. EPA consistently has been given near impossible tasks, unrealistic deadlines, and inadequate funding (ELI 1999; Lazarus 1991; Probst and Sherman 2004).

Historically one of the main reasons for a community or local or state officials to support a Superfund listing was the eligibility to receive federal funding for cleanup through the Superfund. Because the cost of Superfund cleanups has proven to be exorbitant, the eligibility for federal cleanup funds has been imperative in many situations. In the early 1980s, most state agencies handed over sizeable hazardous waste sites to the U.S. EPA without delay. However, since that time many states have developed comprehensive state superfund programs, and often states are able to handle the cleanup of sites without any federal intervention or assistance. Even though state programs have become increasingly proficient at addressing hazardous waste, there are still cases for which the Superfund is an invaluable program, particularly when there are no sources of cleanup funding (i.e., responsible parties) at an expansive cleanup site. Unfortunately, the federal funding behind Superfund has been depleted. The Superfund program was originally financed by a tax levied on the petroleum and chemical industries. However, this tax expired in 1995, and although the fund officially reached a zero balance by the end of 2003, it is partially replenished by cost recovery lawsuits against responsible parties. Additional funds are allocated to Superfund projects from the general fund by congressional appropriations. Total expenditures for Superfund programs have remained between $1.41 billion in 1995 to $1.24 billion in fiscal year 2004 [(U.S. GAO 2004]. Despite the decrease in the federal financing for Superfund projects , a listed Superfund site still has the potential for more funding when compared with the monies available at the state level. Therefore, despite the criticisms levied at the program and how long site cleanups may take, and even though the Superfund program itself has reached a zero balance, the program is still beneficial for sites requiring massive cleanup and/or having no other potential sources for cleanup money. Becoming officially placed on the NPL is a critical step toward acquiring funding necessary for such cleanups.

## Executive Order 12898

Since 1994, when President Clinton signed Executive Order 12898 for environmental justice (Clinton 1994), minority and low-income populations should have been benefiting proportionately from environmental cleanups through the Superfund program. The order specifically demands that agencies, including the U.S. EPA, ensure that their policies and programs do not disproportionately affect minorities and poor. However, whether the agency has complied with this order and to what degree has been questioned.

EPA is essentially relying on state and local governments to deal with the environmental justice concerns…even though the executive order does not apply to state or local governments, and, absent specific state or local law, they have no obligation to consider environmental justice. . . . (U.S. GAO 2005)

Further,

EPA has not fully implemented Executive Order 12898 nor consistently integrated environmental justice into its day-to-day operations. EPA has not identified minority and low-income, nor identified populations addressed in the Executive Order, and has neither defined nor developed criteria for determining disproportionately impacted. (U.S. OIG 2004)

A study examining the executive order as it applies to pesticides in the State of Florida concluded, “the goals of the Executive Order 12898 are not being achieved by the U.S. Environmental Protection Agency” (Murphy-Green and Leip 2002).

Although these reports indicate that the executive order has not been complied with or implemented, many regional U.S. EPA offices are working tirelessly on programs to increase environmental equity. In 2004 the Office of Environmental Justice released a report (U.S. EPA 2005) highlighting regional environmental justice programs and pilot projects. There are a tremendous number of such projects, showing sincere commitment to environmental justice at the regional level. However, without support at the federal level, these programs are left without federal funding and are inconsistent across the nation. Because neither minority nor low-income have been defined at the federal level, regional offices are left attempting to create these definitions on their own and using regional funding to implement programs.

[In] the absence of environmental definitions, criteria, or standards from the Agency, many regional and program offices have taken steps, individually to implement environmental justice policies. This has resulted in inconsistent approaches by regional offices. (U.S. OIG 2004)

In 2001 then U.S. EPA administrator Christine Todd Whitman virtually rewrote Executive Order 12898 and removed the considerations for populations most affected by environmental hazards—minorities and the poor (Whitman 2001). In the memorandum she redefines environmental justice

to mean the fair treatment of people of all races, cultures, and incomes with respect to the development, implementation and enforcement of environmental laws and policies, and their meaningful involvement in the decision making process of the government. (U.S. OIG 2004)

Although advocating fair treatment of all people in regard to environmental laws and policies is certainly commendable, the memorandum is and was not the letter or the spirit of Executive Order 12898. The original wording of the 1994 executive order, required specifically “identifying and addressing” programs that disproportionately affect “minorities and low-income groups.” The order clearly requires agencies to take special consideration of populations that historically have been overburdened with environmental hazards. In contrast, Whitman’s 2001 memorandum indicates a distinct policy shift; agencies are being told they no longer need to consider race, ethnicity, or other socioeconomic indicators in their programs or policies. Although this ‘blind’ justice may seem impartial, these agencies, and the U.S. EPA in particular, were told nearly 7 years before to specifically target populations to ensure proper and proportional treatment. Furthermore, it disregards the historically inequitable treatment and burden that minorities and the poor have born and the reason why the order was constructed initially.

The lack of direction and guidance that appears to be coming from the highest levels at U.S. EPA certainly will have consequences on the ability for regional offices to effectively implement consistent environmental justice programs. Furthermore, it will undoubtedly affect a national evaluation of the Superfund program specifically. In this article I explore how effective Executive Order 12898 has been and what impact it has had on the equitability of Superfund listings.

## Methods

Event history analyzes the risk of an event or hazard, given a set of influencing variables. Environmental cleanup justice research such as that presented in this article seeks to determine if population characteristics influence the Superfund listing process. Using time varying covariates in event history analysis assures that current demographic patterns are not used to explain past Superfund listings. Instead of including independent variable information for only one point in time, event history analysis allows the variable to change over time. This is particularly important for environmental justice research, which has been plagued with the inability to show which came first, hazards or marginalized populations.

The data used in this research are the total population of sites within the United States, in the Superfund program that have reached proposal to the Superfund list by March 2003 (*N* = 1,540) (U.S. EPA 2003). This is the entire set of such sites and is therefore, population-level data. Comparing the chance of listing for sites that have already reached the proposal stage allows for inclusion of HRS. This score is used to help the U.S. EPA decide whether a site qualifies for the Superfund program. However, using all proposed sites can and should be considered a very conservative measure of those at risk of making the Superfund list. There is no doubt a level of power is necessary to have a site officially recognized as discovered by the U.S. EPA, let alone proposed for the Superfund program. Therefore, the conclusions made within should be considered conservative.

Superfund sites were matched to demographic census data by 2000 census tract. The choice of spatial variables in environmental justice or environmental equity studies is always contentious. It is imperative that the unit of analysis accurately portrays the affected community (Bowen 2001; Mohai P, Saha R, unpublished data, 2003). Within traditional environmental justice research, arguments about spatial units are most often in reference to or questioning whether or chosen unit is truly an exposed population; this is not an issue in this present study. Here, the unit of analysis is the group that generally surrounds the site and is also considered to be or perceived as being affected by the site, not those that are exposed such as in traditional environmental justice research. Therefore, although a county would be much too large, a census block would be far too small and could also contain a greater variation in population size. The unit that is most appropriate would be the ZIP code or census tract closest to the site. In this case, we have chosen census tract. Again, this should be considered a conservative choice. Support for the choice of census tracts as the unit of analysis is found throughout environmental justice and Superfund research (Anderton et al. 1994a, 1994b, 1997; Been 1994; Been and Gupta 1997; Boer et al. 1997; Bowen and Haynes 2000a, 2000b; Feldmen and Hanahan 1996; Mitchell et al. 1999; Stretesky and Hogan 1998; Stretesky and Lynch 1999; Yandle and Burton 1996).

Because the 2000 census tract was available for each of the sites in the Superfund data set, it was the starting point for census data. The demographic census data used for this analysis are historical census data; current 2000 census tract delineations are traced backward in time using Geolytics software (http://www.geolytics.com) and the NCDB (Neighborhood Change Database; http://geolytics.com/USCensus,Neighborhood-Change-Database-1970–2000,Products.asp). Event history analysis requires that independent time-varying covariates change at the same interval as the dependent variable. This means that independent demographic census variables that change over time, such as percentage minority or mean family income, must be measured in the same time intervals as the dependent variable—U.S. EPA Superfund listing decisions. A yearly time interval was chosen as the most appropriate interval estimation of Superfund listings. Therefore, this analysis uses interpolated census data from these three decades, creating yearly census data for each case.

Variables analyzed in this research are both demographic and site specific. Demographic variables are taken from the U.S. Census. Site-specific variables are taken from the U.S. EPA’s Comprehensive Environmental Response and Liabilities Information System (CERCLIS) database (U.S. EPA 2003). Demographic variables are split into three groupings: marginalized populations, wealth and power indicators, and gender and family. Because studies suggest that minority, low-income, and less-educated groups are disproportionately exposed to or in closer proximity to environmental pollutants, the associations between hazardous waste and other marginalized populations should be similarly explored. Therefore, marginalized population variables include percent Hispanic, Native American, minority, institutionalized, and elderly. Indicators of wealth, status, and power include mean family income, mean housing values, percent owned housing, percent with a college degree or higher, percent employed in professional careers, percent with no high school diploma, and percent of families below the poverty line.

Superfund equity research has not thoroughly examined the relationship between gender and environmental remediation. Therefore, the third grouping of variables focuses on gender and family variables. Environmental justice literature indicates that women and people of color have historically fueled the grassroots activism of the environmental justice movement (Brown and Mikkleson 1997; Bullard 1990, 1993; Cable 1992; Edelstein 1988; Faber 1998; Levine 1982). Additionally, research focusing on gender and the environment indicates that women display greater concerns regarding local environmental hazards (Blocker and Eckberg 1989; Davidson and Freudenburg 1996; Flynn et al. 1994; Krauss 1993; Stern et al. 1993; Van Liere and Dunlap 1980). This research implies that women exhibit more concern over local environmental hazards because of their role as caregivers and nurturers, which also implies a distinct relationship between environmental hazards and children, one in which children are protected from such hazards. Therefore, grassroots environmental justice, gender, and family indicators include percent of female-headed households and children. To control for the increases in industrialization that often occurred historically in highly populated city centers, two indicators of urbanization were included. The first is an urbanization indicator created by the U.S. census, which measures the percent of a tract living in an urban center (this is the only demographic variable that is does not vary over time; the value for the year 2000 is used for each case). The second, population density, is the number of persons (thousands) per square mile. Site-specific variables explored in this analysis include identification of Potentially Responsible Parties (PRPs), and the HRS of the site.

## What Are the Chances of Making the Superfund List?

Generally, inclusion on the Superfund list does not appear as likely for sites in areas with high minority and poor populations (Table 1). Increases in minority populations, families in poverty, or people without high school diplomas all lower the chances of a Superfund listing. The data indicate that a 1% increase in minority is associated with a 0.2% decrease in the chance of a Superfund listing. Using the (Exp (b)-1) × 100 method, a multiplicative method, therefore indicates that a 10% higher minority population lowers the chance of a Superfund listing by 2%, whereas a 10% higher poverty rate lowers the chance of being listed by 13%. Conversely, a site with a $10,000 higher mean income has a 9% greater chance of making the Superfund list (note the variable was measured in thousands). These results further indicate that minorities and the poor are not benefiting from Superfund cleanups, whereas wealthier communities have a greater chance of obtaining this benefit.

Results for Hispanic and Native Americans, more specifically, are not the same. Sites in areas with higher Hispanic or Native American populations actually have greater chances of being placed on the Superfund list. Sites in areas with high percentages of female-headed households have greater chances of being listed. Because women, and specifically women of color, have been a significant force in the grass-roots environmental justice movement, this is a particularly interesting finding. Whether these women have had a great impact on the Superfund program cannot be discerned from these data, but considering these women may otherwise be considered a marginalized population, it is interesting to find this population has a greater chance of Superfund listings.

Site-specific variables also help in identifying the chance of a Superfund listing. Specifically, the identification of a PRP who would be forced to contribute to cleanup costs if able increases the chances of a Superfund listing by roughly 1.3. A site with an HRS of 10 points higher has a 22% lower chance of being listed.

## Has Executive Order 12898 Rectified Superfund Equity?

To evaluate the impact of Executive Order 12898 on Superfund listings pre- and post-executive order, Superfund sites were tested using the previous model. The data set was split into sites discovered before 1994 and sites discovered in 1994 or later. The number of sites discovered in 1994 or later is small (61 cases), but this should not affect the conclusions drawn from this group, as these are population data. However, this should encourage further research into these trends when more data are available.

What is most unsettling from this comparison is that the chance of listing for several marginalized and poor populations actually worsens for sites discovered since the executive order (Table 2). For example, for sites discovered in 1994 or later, a 10% higher minority population lowers the chance of listing 7% (compared with 3% for early sites), and a 10% higher Native American population lowers the chance of a site making the list by almost 80% (compared with a 3% greater chance pre-executive order). Poorer populations are also less likely to be placed on a Superfund list after the executive order; a 10% increase in poverty decreases the chances of listing by 31% after the executive order (as opposed to 16% before the executive order).

Conversely, increases in income continue to be associated with greater chances of Superfund listing. Housing becomes an even stronger indicator of the chance of listing; increases in housing values and housing ownership indicate an increased chance of Superfund listing post-executive order. Other indicators of wealth are, at best, mixed for sites discovered in 1994 or later. For example, for sites discovered in 1994 or later, college educations and professional careers become associated with a decreased chance of making the list.

Another interesting finding post-executive order, is that higher percentages of people without high school diplomas actually increases the chance of listing. Other indicators such as lower incomes and lower housing values as well as higher poverty rates are associated with a lower chance of listing, whereas sites with greater percentages of people without high school diplomas increase the chance. It is possible that Superfund listings are more likely in areas where homeowners are less educated or are considered working class. This is supported to varying degrees by previous research, which indicated that both the wealthiest and poorest populations were not represented in Superfund and that Superfund is a working-class program (Anderton et al. 1997; Hird 1993; O’Neil 2005). It is possible that more powerful communities may attempt to rectify or remediate hazardous waste using alternative means such as redevelopment projects or state programs.

## Discussion

Evidence further supports an environmental cleanup justice theory, suggesting that marginalized and poor populations are less likely to benefit from a cleanup program such as Superfund despite their overrepresentation in proximity to environmental hazards. These data indicate that even if sites in minority and poor areas do make it into the Superfund program, they have less chance of making the official Superfund list. Being placed on the official Superfund list is a critical step in securing funding for hazardous sites, especially as these sites are now financed through Senate appropriations. Sites not officially listed in the program will have significant difficulty obtaining necessary funding from the government. For sites with exorbitant cleanup costs and no other potential funding sources, listing on the Superfund is still critical.

Past research-excluding factors such as the HRS and PRPs may have missed important aspects of the Superfund process. Despite that the U.S. EPA uses the HRS only to qualify sites for the NPL and not for a literal ranking, HRS appears to be one of the strongest predictors of a Superfund listing. It is possible that sites with high HRSs are sites that also often require intensive negotiation with PRPs. Extremely hazardous sites with a viable PRP may become enmeshed in litigation and negotiation, thereby taking much longer to list. It is also possible that very hazardous sites are also more difficult (and take longer) to analyze, create a hazard score for, and prepare for proposal and therefore listing. Alternatively, previous research has indicated, “EPA is more likely to tackle “easier” or low-risk sites” (Daley and Layton 2004). These authors theorized that tackling the lower-risk sites was a pragmatic approach for an agency faced with serious financial constraints and much criticism in terms of results. These data further support this theory and suggest that less hazardous sites have a greater chance of reaching an official Superfund listing.

Perhaps more important, the use of HRS controls for the hazardousness of the site in this analysis. Therefore, although it could be argued that perhaps sites in minority and poor areas are not making the list because they are not as hazardous, this analysis controls for just that fact. Furthermore, because the data set includes sites already proposed for Superfund listing, the sites have all qualified for the Superfund in terms of their hazard.

The relationship between the chance of listing and the identification of a PRP is complex. Results show an inconsistent relationship and suggest further research into the nuances of this association. For all sites in the data set, and for the first 13 years of Superfund specifically, the identification of a PRP actually increased the chance of a Superfund listing. Because of the severe financial constraints on the Superfund program and the exorbitant costs of cleanups coupled with inadequate funding, it is possible that sites with PRPs move through the program more quickly because of the potential for external funding. A site is placed on the official Superfund list has legal benefits through the long reach of the Superfund’s joint and several liabilities clause. This clause states that parties responsible for waste at a Superfund site can be held liable even if they did not intend to pollute and that all parties that handled the waste can be pursued. The percentage of sites cleaned using funds from PRPs is estimated as high as 70% (U.S. EPA 2000). Therefore, sites with PRPs may be easier to list because they may be perceived as not competing for the scarce cleanup resources provided by the Superfund.

However, for sites discovered since 1994, the opposite was found; sites with identified PRPs have a lower chance of reaching official Superfund listing designation. There are several possible reasons why a site with a PRP may take longer or have a lower chance to reach the list. First, a PRP may wish to contest the listing process to avoid the stigma and financial burden that comes with a Superfund label. Furthermore, a company could enter into an agreement with the U.S. EPA to clean the site, with the understanding that the U.S. EPA will not put the site on the official Superfund list. This would cause a site to remain in the proposal stage until the company and the U.S. EPA decide that the site is clean, lowering the chance of reaching listing. These results may indicate that PRPs now more than ever avoid or attempt to avoid Superfund and negotiate and pay for cleanups outside the boundaries of the Superfund program. However, the complex relationship between PRPs and listing cannot be fully established with these data, and further research is encouraged.

It was anticipated that the implementation of environmental justice programs within the U.S. EPA in the mid-1990s, and Executive Order 12898 would lessen the degree to which demographic factors were associated with the chance of listing. However, the results of the present study show the opposite; in general, the results further support the conclusion that sites discovered in 1994 or later put minorities and the poor at even lower chances of listing than in previous years—a conclusion that is quite controversial considering the specific requirements of Executive Order 12898. It is possible that the benefits of environmental justice programs implemented in the wake of the order are not yet showing in the results of this study, or that the addition of new sites will show a more equitable trend. It is also possible that the U.S. EPA has focused environmental justice efforts toward other programs such as the Brownfield cleanups. Research has shown that the Brownfield program does appear to be incorporating environmental justice principles (Solitare and Greenberg 2002). This, however, does not mitigate the fact that Superfund listings for sites in minority and low-income areas are even less likely since enactment of environmental justice legislation. Sites proposed for Superfund listing cannot qualify for the Brownfield program because of their level of hazard; the Brownfield program is for sites with lower levels of hazard. However, it is possible that institutional controls or other potential site reuses are being preferred over cleanup in poorer communities. Because these reuses have the potential to revitalize a community through increased tax revenues and job creation, it is possible they may be encouraged in poor communities instead of traditional Superfund cleanups. Last, it is possible that the U.S. EPA has chosen to focus on the cleanup of Superfund sites rather than the equitable listing of such sites. Nearly 80% of all deleted sites have been deleted since 1994 (214 of 269) (O’Neil 2005).

## Conclusions

The results of the present study suggest that for sites discovered after the 1994 Executive Order 12898, there is even a lesser chance for Superfund listing for marginalized and poor populations. Despite legislation to ensure environmental justice, equity in Superfund listing appears to be worsening. It appears that the U.S. EPA has failed to consistently implement the executive order regarding the Superfund program. There have been numerous regional successes, but on the national level, the Superfund program has not complied with the executive order. Furthermore, because the regionally specific implementation of the order, there is reason to believe that results separated by U.S. EPA region would differ significantly. For this reason, further research is necessary to focus on the comparison of U.S. EPA regional office practice and institutionalization of the order as well as regional discrepancies regarding the success of the order in establishing equitable Superfund listings.

These results are not surprising because of the guidance and funding that regional offices have been given pertaining to Executive Order 12898. Regional offices were instructed to comply with the order, but the order fails to define what disproportionate treatment is or how to measure it. In addition, the order fails to define minority or low income that has caused regional discrepancies. Furthermore, the Office of Environmental Justice, “does not provide funding, and has no authority over the program and regional offices regarding efforts to integrate environmental justice” (U.S. OIG 2004). The commitment to environmental justice at the highest levels of the U.S. EPA could be described as inconsistent and even contradictory. The words of one U.S. EPA administrator in effect redefined the goal of environmental justice. Clearly, the U.S. EPA has incorporated this new redefinition of environmental justice as it responded to criticism about environmental justice by noting,

The Agency does not take into account the inclusion of the minority and low-income populations, and indicated it is attempting to provide environmental justice for everyone. (U.S. OIG 2004)

The U.S. EPA needs to recognize the original intent of Executive Order 12898 and reestablish the environmental justice mission. The order expressly notes that,

each Federal agency shall conduct its programs policies and activities that substantially affect human health or the environment, in a manner that ensures that such programs, policies and activities do not have the effect of excluding persons (including populations) from participation in, denying persons (including populations) the benefits of, or subjecting persons (including populations) to discrimination under, such, programs, policies, and activities because of their race, Color or national origin. (U.S. OIG 2004)

The order requires that

each Federal agency shall make achieving environmental justice part of its mission by identifying and addressing, as appropriate, disproportionately high and adverse human health or environmental effects of its programs, policies, and activities on minority populations, and low-income populations. . . . (U.S. OIG 2004)

The Superfund program has both excluded persons from listings and denied persons the benefit of a cleaner environment, and therefore violated the order.

If Executive Order 12898 is to be complied with properly, it needs not only increased financial support but also support through consistency of definition and implementation. Furthermore, the order would be more powerful if there were repercussions from the lack of its implementation. Regional offices work aggressively to increase environmental equity but are doing so with inconsistent and sometimes negative federal support and minimal federal funding. Without proper implementation, these programs will have trouble making the impact necessary to rectify the problem.

## Figures and Tables

**Table 1:** Chance of a Superfund listing for all sites proposed to Superfund.

Cox proportional hazard ratios	All sites [Exp(B)]	Chance of listing (%)^a^
Marginalized populations		
Hispanic (%)	1.004	4
American Indian (%)	1.002	2
Minority (%)	0.998	−2
Institutionalized population (%)	1.002	2
Elder population (%)	1.001	1
Wealth and power indicators		
Mean family income ($1,000s)	1.009	9
Mean value of housing ($1,000s)	0.999	−1
Owned housing (%)	1.001	1
Professional careers (%)	1.000	0
College degree or higher (%)	1.000	0
No high school diploma (%)	0.998	−2
Below the poverty line (%)	0.987	−13
Gender and family		
Female headed households (%)	1.007	7
Children < 18 (%)	1.000	0
Urbanization		
Population density	1.000	0
Urban	0.998	−2
Site-specific characteristics		
PRPs identified	1.308	131
Hazardous ranking score	0.978	−22
Cases		
Listed	1,463	
Censored	56	
Total	1,519	
Missing	21	

Exp(B), Cox proportional hazard ratios derived from Cox proportional hazards method (coefficients not shown).

a Chance of listing defined using the multiplicative method (Exp (b)-1) × 100. The chance of listing is calculated using a 10% increase in (%) variables. Variables not calculated as percentages (income or housing) are associated with a $10,000 increase, whereas Hazard Score is associated with a 10-point increase in Hazard Score, and finally PRP, if the chance of listing is in relation to the identification of a PRP as opposed to not identifying one.

**Table 2:** Chance of a Superfund listing before and after Executive Order 12898.

Cox proportional hazard ratios	Sites discovered before E.O. [Exp(B)]	Chance of listing (%)^a^	Sites discovered after E.O. Exp(B)	Chance of listing (%)^a^
Marginalized populations				
Hispanic (%)	1.002	2	1.005	5
American Indian (%)	1.003	3	0.923	−77
Minority (%)	0.998	−2	0.993	−7
Institutionalized population (%)	1.002	2	0.998	−2
Elder population (%)	0.999	−1	1.042	42
Wealth and power indicators				
Mean family income ($1,000s)	1.004	4	1.004	4
Mean value of housing ($1,000s)	0.999	−1	1.006	6
Owned housing (%)	1.001	1	1.019	19
Professional careers (%)	1.007	7	0.934	−66
College degree or higher (%)	1.001	1	0.975	−25
No high school diploma (%)	1.000	0	1.010	10
Below the poverty line (%)	0.984	−16	0.969	−31
Gender and family				
Female headed households (%)	1.005	5	1.048	48
Children < 18 (%)	1.000	0	0.991	−9
Urbanization				
Population density	1.000	0	1.000	0
Urban	0.998	−2	1.002	2
Site-specific characteristics				
PRPs identified	1.344	134	0.617	62
Hazardous ranking score	0.977	−23	0.987	−13
Cases				
Listed	1,410		53	
Censored	48		8	
Total	1,458		61	
Missing	21		0	

Abbreviations: Exp(B), Cox proportional hazard ratios derived from Cox proportional hazards method (coefficients not shown). E.O., executive order.

a Chance of listing defined using the multiplicative method (Exp (b)-1) × 100. The chance of listing is calculated using a 10% increase in (%) variables. Variables not calculated as percentages (income or housing) are associated with a $10,000 increase, while Hazard Score is associated with a 10-point increase in Hazard Score, and finally PRP, if the chance of listing is in relation to the identification of a PRP as opposed to not identifying one.
