# Erratum

**Published:** 2007-08

In the article by O’Neil [ Environ Health Perspect 115:1087–1093 (2007)], the corresponding author’s address is incorrect. The correct address is S.G. O’Neil, 1071 Blue Hill Ave., Milton, MA 02186. Telephone: 617-333-0500. E-mail: soneil0905@curry.edu

*EHP* regrets the error.

